# An alphavirus-derived replicon RNA vaccine induces SARS-CoV-2 neutralizing antibody and T cell responses in mice and nonhuman primates

**DOI:** 10.1126/scitranslmed.abc9396

**Published:** 2020-07-20

**Authors:** Jesse H. Erasmus, Amit P. Khandhar, Megan A. O’Connor, Alexandra C. Walls, Emily A. Hemann, Patience Murapa, Jacob Archer, Shanna Leventhal, James T. Fuller, Thomas B. Lewis, Kevin E. Draves, Samantha Randall, Kathryn A. Guerriero, Malcolm S. Duthie, Darrick Carter, Steven G. Reed, David W. Hawman, Heinz Feldmann, Michael Gale, David Veesler, Peter Berglund, Deborah Heydenburg Fuller

**Affiliations:** 1 Department of Microbiology, University of Washington, Seattle, WA 98109.; 2 HDT Bio Corp, Seattle, WA 98102.; 3 PAI Life Sciences, Seattle, WA 98102.; 4 Laboratory of Virology, Division of Intramural Research, National Institute of Allergy and Infectious Diseases, National Institutes of Health, Hamilton, MT 59840.; 5 Center for Innate Immunity and Immune Disease, University of Washington, Seattle, WA 98109.; 6 Department of Biochemistry, University of Washington, Seattle, WA 98195.; 7 Washington National Primate Research Center, Seattle, WA 98121.; 8 Department of Immunology, University of Washington, Seattle, WA 98109.

## Abstract

The COVID-19 pandemic, caused by infection with the SARS-CoV-2 coronavirus, is having a deleterious impact on health services and the global economy, highlighting the urgent need for an effective vaccine. Such a vaccine would need to rapidly confer protection after one or two doses and would need to be manufactured using components suitable for scale-up. Here, we developed an alphavirus-derived replicon RNA vaccine candidate, repRNA-CoV2S, encoding the SARS-CoV-2 spike (S) protein. The RNA replicons were formulated with Lipid InOrganic Nanoparticles (LION) that were designed to enhance vaccine stability, delivery, and immunogenicity. We show that a single intramuscular injection of the LION/repRNA-CoV2S vaccine in mice elicited robust production of anti-SARS-CoV-2 S protein IgG antibody isotypes indicative of a Type 1 T helper cell response. A prime/boost regimen induced potent T cell responses in mice including antigen-specific responses in lung and spleen. Prime-only immunization of aged (17-month old) mice induced smaller immune responses compared to young mice, but this difference was abrogated by booster immunization. Importantly, in nonhuman primates, prime-only immunization in one intramuscular injection site or prime/boost immunizations in 5 intramuscular injection sites elicited modest T cell responses and robust antibody responses. The antibody responses persisted for at least 70 days and neutralized SARS-CoV-2 at titers comparable to those in human serum samples collected from individuals convalescing from COVID-19. These data support further development of LION/repRNA-CoV2S as a vaccine candidate for prophylactic protection against SARS-CoV-2 infection.

## Teaser

A prime-only replicating RNA vaccine delivered with lipid inorganic nanoparticles induces neutralizing antibody and T cell responses against SARS-CoV-2 in aged mice and macaques.

## INTRODUCTION

Severe acute respiratory syndrome coronavirus-2 (SARS-CoV-2) first emerged in December 2019 and within 3 months, Coronavirus Disease 2019 (COVID-19), caused by SARS-CoV-2 infection, was declared a worldwide pandemic (*1*–*3*). Coronaviruses are enveloped, single-strand positive-sense RNA viruses with a large genome and open reading frames for four major structural proteins: Spike (S), envelope, membrane, and nucleocapsid. The S protein mediates binding of coronaviruses to angiotensin converting enzyme 2 (ACE2) on the surface of various cell types including epithelial cells of the pulmonary alveolus (*4*–*6*). Protection is thought to be mediated by neutralizing antibodies against the S protein (*7*, *8*), as most of the experimental vaccines developed against the related SARS-CoV incorporated the S protein, or its receptor binding domain (RBD), with the goal of inducing robust, neutralizing antibody responses (*9*–*11*). Indeed, previous reports have shown that human neutralizing antibodies protected mice challenged with SARS-CoV (*12*–*14*) and Middle East respiratory syndrome (MERS)-CoV (*15*) suggesting that protection against SARS-CoV-2 could be mediated through anti-S antibodies. Additionally, SARS vaccines that drive Type 2 T helper cell (Th2) responses have been associated with enhanced lung immunopathology following challenge with SARS-CoV, whereas those with a Type 1 T helper cell (Th1)-biased immune response are associated with enhanced protection in the absence of lung immunopathology (*16*, *17*). Therefore, an effective COVID-19 vaccine will likely need to induce, at the very least, Th1-biased immune responses comprised of SARS-CoV-2-specific neutralizing antibodies.

Nucleic acid vaccines have emerged as ideal modalities for rapid vaccine design, requiring only the target antigen’s gene sequence. Other advantages include no need for pathogen culture (inactivated or live attenuated vaccines) or scaled recombinant protein production. In addition, nucleic acid vaccines avoid the problem of pre-existing immunity that can dampen immunogenicity of viral vector vaccines. Recently, clinical trials were initiated with messenger RNA (mRNA) vaccines formulated with lipid nanoparticles (LNPs) and a DNA vaccine delivered by electroporation (*18*). However, mRNA and DNA vaccines may not be able to induce protective efficacy in humans after a single immunization since, similar to inactivated and recombinant subunit protein vaccines, they typically require multiple administrations over an extended period of time to become effective (*19*). Virus-derived replicon RNA (repRNA) vaccines were first described in 1989 and have been delivered in the forms of virus-like RNA particles (VRP), *in-vitro* transcribed (IVT) RNA, and plasmid DNA (*20*–*23*). In repRNA vaccines, the open reading frame encoding the viral RNA polymerase complex (most commonly from the *Alphavirus* genus) is intact but the structural protein genes are replaced with an antigen-encoding gene (*20*, *24*–*26*). Whereas conventional mRNA vaccines, like those initiated in recent clinical trials, are translated directly from the incoming RNA molecules, introduction of repRNA into cells initiates ongoing biosynthesis of antigen-encoding RNA that results in dramatically increased expression and duration that enhances humoral and cellular immune responses (*27*). In addition, repRNA vaccines mimic an alphavirus infection in that viral-sensing stress factors are triggered and innate pathways are activated through Toll-like receptors and retinoic acid inducible gene (RIG)-I to produce interferons, pro-inflammatory factors and chemotaxis of antigen-presenting cells, as well as promoting antigen cross-priming (*28*). As a result, repRNA acts as its own adjuvant, eliciting more robust immune responses after a single dose, relative to conventional mRNA which typically requires multiple, 1,000-fold higher doses (*29*). An effective vaccine to stop a pandemic outbreak like COVID-19 would ideally induce protective immunity rapidly and after only a single dose thus reducing the load on manufacturing at scale due to a requirement for fewer and lower doses. Given that repRNA vaccines often require only a single administration to be effective (*30*), they offer considerable potential to meet this need.

## RESULTS

### Vaccine design and formulation

**Fig. 1:**
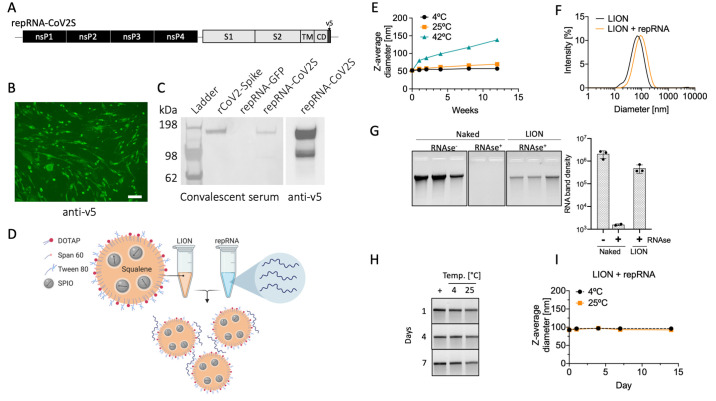
repRNA-CoV2S vaccine design and formulation. (**A**) Shown is the codon-optimized full length spike (S) protein open reading frame, including the S1, S2, transmembrane (TM), and cytoplasmic (CD) domains, corresponding to positions 21,536 to 25,384 in the S protein of SARS-CoV-2 isolate Wuhan-Hu-1 (GenBank: MN908947.3). This construct was fused to a C-terminal v5 epitope tag and then was cloned into an alphavirus replicon encoding the 4 nonstructural protein (nsP1-4) genes of Venezuelan equine encephalitis virus, strain TC-83. Following RNA transcription and capping, repRNA-CoV2S was transfected into BHK cells. 24 hours later, the transfected BHK cells were analyzed by (**B**) anti-v5 immunofluorescence and (**C**) Western blot using either convalescent human serum or anti-v5 serum for immunodetection. Recombinant SARS-CoV2 spike protein (rCoV2-Spike) and repRNA-GFP were used as positive and negative controls, respectively. (**D**) Shown is a graphical representation of LION and formation of the vaccine complex after mixing with repRNA. (**E**) Shown is the evolution of LION particle size over 15 weeks measured by dynamic light scattering during storage at 4°C, 25°C or 42°C. (**F**) After mixing LION particles and repRNA, complex formation was confirmed by a shift in size distribution. (**G**) Gel electrophoresis analysis of triplicate preparations of repRNA extracted from LION particles after a concentrated RNase challenge showed substantial protection relative to a triplicate preparation of a dose-matched naked RNA following RNAse challenge. The formulated vaccine was stable for at least one week after mixing and storage at 4°C or 25°C as determined by (**H**) gel electrophoresis of repRNA extracted by phenol-chloroform treatment and (**I**) particle size of the complex. Data in **B** and **C** are representative of 2 independent experiments. Data in **E**, **H**, and **I** are from a single experiment, whereas data in **F** and **G** are representative of 3 independent experiments. Data in **E, G,** and **I** are shown as mean ± s.d. of 3 technical replicates. Scale bar in **B** is 100 μm.

Building on experience with the attenuated Venezuelan equine encephalitis virus (VEEV) TC-83 strain (*22*, *30*–*34*), we generated repRNAs incorporating sequences from the SARS-CoV-2 Spike (S) protein, including full length S (repRNA-CoV2S) (Fig. 1A). Using immunofluorescence and Western blot we demonstrated efficient expression of the v5-tagged S protein in BHK cells (Fig. 1B,C). Then, utilizing convalescent serum collected 29 days after onset of COVID-19 as an immunodetection reagent, we demonstrated endogenous expression of S protein in BHK cells, reactive with natural SARS-CoV-2 immune sera (Fig. 1C). Next, we evaluated the ability of repRNA-CoV2S to rapidly generate antibody and T cell responses in mice when formulated with a Lipid InOrganic Nanoparticle (LION) emulsion designed to enhance vaccine stability and intracellular delivery of the vaccine.

LION is a highly stable cationic squalene emulsion with 15 nm superparamagnetic iron oxide (Fe_3_O_4_) nanoparticles (SPIO) embedded in the hydrophobic oil phase. Squalene is a known vaccine adjuvant (*35*, *36*) and SPIO nanoparticles have a long history of clinical use in MRI contrast and intravenous iron replacement therapy; the unique nonlinear magnetic properties of SPIOs have also been leveraged for new uses in a range of imaging, targeting and therapy applications (*37*–*42*). A key component of LION is the cationic lipid 1,2-dioleoyl-3-trimethylammonium propane (DOTAP), which enables electrostatic association with RNA molecules when combined by a simple 1:1 (v/v) mixing step (Fig. 1D). LION has an intensity-weighted average diameter of 52 nm (PDI = 0.2) measured by dynamic light scattering. The formulation is colloidally stable for at least 3 months when stored at 4 and 25°C (Fig. 1E). When mixed, electrostatic association between anionic repRNA and cationic DOTAP molecules on the surface of LION promotes immediate complex formation, as confirmed by an increase in particle size to an intensity-weighted average diameter of 90 nm detected by dynamic light scattering (Fig. 1F). Gel electrophoresis analysis of LION-formulated repRNA molecules extracted by phenol-chloroform treatment after a concentrated RNase challenge showed substantial protection from RNase-catalyzed degradation compared to unformulated repRNA (Fig. 1G). To evaluate short-term stability of the vaccine, we evaluated repRNA integrity and complex stability on 1, 4 and 7 days after mixing. LION maintained full integrity of the repRNA molecules (Fig. 1H) and complex size (Fig. 1I) at all time points.

### Immunogenicity of LION/repRNA-CoV2S in C57BL/6 mice

**Fig. 2:**
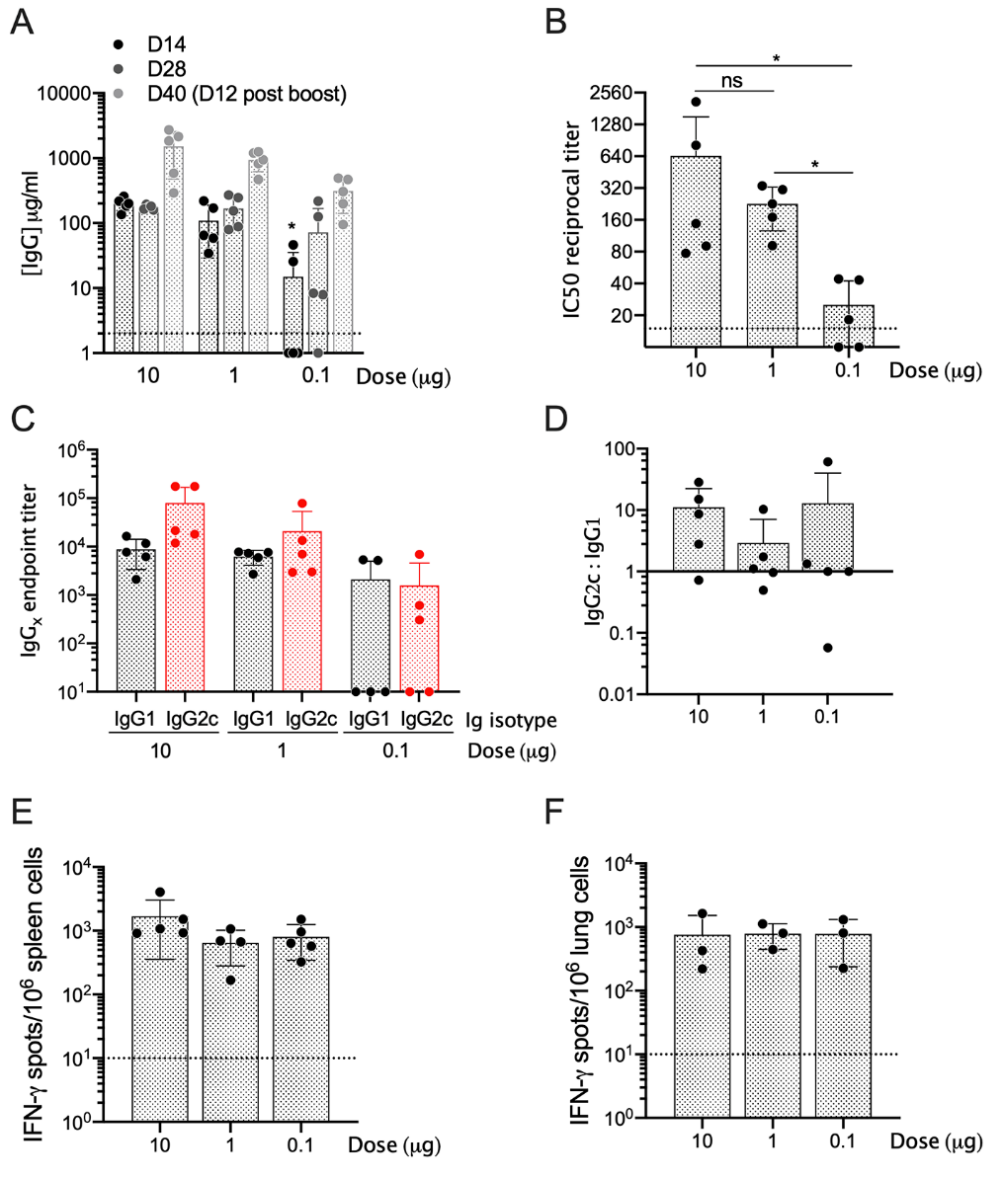
The LION/repRNA-CoV2S vaccine induces Th1-biased and neutralizing antibodies in C57BL/6 mice. Six to eight-week old C57BL/6 mice (n=5/group) received 10, 1, or 0.1 μg LION/repRNA-CoV2S via the intramuscular route on days 0 and 28. (**A**) Anti-S IgG antibody concentrations were determined by enzyme linked immunosorbent assay (ELISA) on days 14, 28, and 40. For day 14 samples, (**B**) 50% inhibitory concentrations (IC50) were determined by pseudovirus (SARS-CoV-2 Wuhan-Hu-1 pseudotype) neutralization assays. For day 14 samples, (**C**) anti-S IgG1 and IgG2c antibody endpoint titers and (**D**) ratios were determined by ELISA. On day 40, 12 days after a booster immunization, (**E**) spleens and (**F**) lungs were harvested and IFN-γ responses were measured by enzyme-linked immune absorbent spot (ELISpot) assay following an 18-hour stimulation with 10 peptide pools encompassing the S protein and consisting of 15 mers overlapping by 11 amino acids (see Fig. S1). Data in **A**, **C**, and **D** are representative of 3 independent experiments; data in **B**, **E**, and **F** are from a single experiment. Dotted lines in **A, B, E,** and **F** represent the lower limit of detection. All data are represented as individual values as well as mean ± s.d. *p<0.05 as determined by one-way ANOVA with Tukey’s multiple comparison test.

A single site intramuscular immunization of C57BL/6 mice (n=5/group) with 10 or 1 μg of LION/repRNA-CoV2S induced 100% seroconversion by 14 days post-immunization and robust anti-S IgG with mean binding concentrations of 200 and 109 μg/ml, respectively, and partial seroconversion (2 out of 5) at a 0.1 μg dose, increasing to 4 out of 5 by day 28 (Fig. 2A). All three dose groups, 10, 1, and 0.1 μg, responded to a boost immunization, reaching anti-S mean IgG concentrations of 1517, 937, and 312 μg/ml, respectively (Fig. 2A). Both the 10 and 1 μg prime-only doses induced neutralizing antibodies with mean 50% inhibitory concentrations (IC50) of 1:643 and 1:226, respectively, significantly higher than titers induced by the 0.1 μg dose (p=0.03 and p=0.02, respectively), as measured by the pseudovirus neutralization assay (SARS-CoV-2 Wuhan-Hu-1 pseudotype) (Fig. 2B). While all doses induced Th1-biased immune responses indicated by higher IgG2c responses when compared to IgG1 (Fig. 2C), there was a trend toward higher doses inducing greater Th1-biased responses as indicated by higher IgG2c:IgG1 ratios (Fig. 2D). Given the potential role for T cells to contribute to immune protection, as seen with SARS and MERS (*43*–*45*), especially in the presence of waning antibody and memory B cell responses, we also evaluated T cell responses to LION/repRNA-CoV2S in mice. On day 28 this same cohort of mice received a second immunization and 12 days later, spleens and lungs were harvested and stimulated with an overlapping 15-mer peptide library of the S protein; IFN-γ responses were measured by enzyme-linked immune absorbent spot (ELISpot) assay. Mice receiving a 10, 1, or 0.1 μg prime/boost exhibited robust splenic T cell responses with mean IFN-γ spots/10^6^ cells of 1698, 650, and 801, respectively (Fig. 2E). Robust T cell responses were also detected in the lung and were similar between groups with mean IFN-γ spots/10^6^ cells of 756, 784, and 777, respectively (Fig. 2F). Interestingly, analysis of the specificity of peptide response showed a biased response toward the S1 domain of the S protein in the spleen (Fig. S1A), whereas responses in the lung were more broadly distributed between the S1 and S2 domains of the S protein (Fig. S1B).

### Immunogenicity of LION/repRNA-CoV2S in young and aged BALB/c mice

**Fig. 3:**
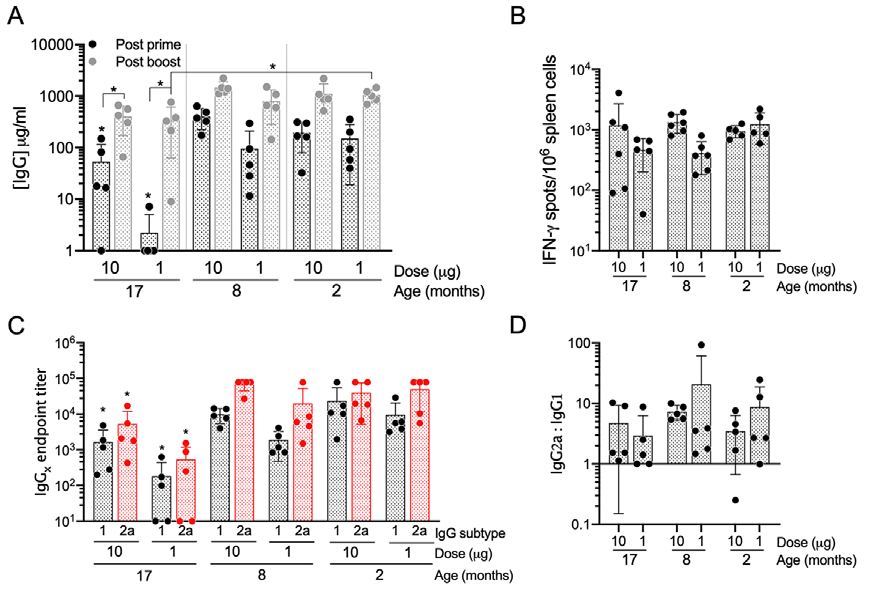
LION/repRNA-CoV2S induces Th1-biased antibodies in aged BALB/c mice. Two-, eight-, or seventeen-month old BALB/c mice (n=5/group) received 10 or 1 μg LION/repRNA-CoV2S via the intramuscular route on days 0 and 28. On day 14 after prime and day 12 after boost (**A**) anti-S IgG was measured by ELISA. On day 40, 12 days after the boost, spleens were harvested and (**B**) IFN-γ responses were measured by enzyme-linked immune absorbent spot (ELISpot) assay following an 18 hour stimulation with 10 peptide pools encompassing the S protein and consisting of 15 mers overlapping by 11 amino acids (see Fig. S1). (**C**) Anti-S IgG1 and IgG2a antibody endpoint titers and (**D**) ratios were determined by enzyme-linked immunosorbent assay (ELISA) 14 days after the prime immunization. Data in 17-, 8-, and 2-month old BALB/c mice are from a single experiment; data for the 2-month old BALB/c mice were replicated in a second experiment. All data are represented as individual values as well as mean ± s.d. *p<0.05 as determined by one-way ANOVA with Tukey’s multiple comparison test between the 17-month old animals and either the 8- or 2-month old animals.

The elderly are among the most vulnerable to COVID-19, but immune senescence in this population poses a barrier to effective vaccination. To evaluate the effect of immune senescence on immunogenicity, we next administered 10 or 1 μg of LION/repRNA-CoV2S in 2-, 8-, and 17-month old BALB/c mice and measured anti-S IgG concentrations at 14 days after prime and 12 days after a booster immunization. Significantly lower antibody responses were observed in the 17-month old mice at both doses following a prime immunization (Fig. 3A), when compared to 2- and 8-month old mice (p=0.001 and p=0.03, for the high dose, respectively, and p<0.0001 at both age groups for the low dose). However, following a boost immunization, the 17-month old mice developed significantly higher antibody responses relative to their prime responses (p=0.001 and p<0.0001, for the 10 and 1 μg doses, respectively), approaching the post-boost responses seen in the 8- and 2-month old mice; antibody responses in the 17-month old 1 μg dose group were still significantly lower than the 2-month-old counterpart (p=0.05) (Fig. 3A). No differences were observed between the 2- and 8-month old mice (Fig. 3A). At day 12 following the boost immunization, spleens were harvested and stimulated with an overlapping 15-mer peptide library of the S protein, and IFN-γ responses were measured by ELISpot assay (Fig. 3B). Mice receiving a 10 or 1 μg prime/boost exhibited robust splenic T cell responses with mean IFN-γ spots/10^6^ cells of 1179, 1328, and 946 in the 10 μg groups and 458, 409, and 1231 in the 1 μg groups for the 17-, 8-, and 2-month old mice, respectively (Fig. 3B). No significant differences were observed between age groups, however, more variability in T cell responses were observed in the 17-month old mice relative to the 8- and 2-month old mice. Interestingly, although BALB/c mice tend to develop a more Th2 immune-biased response following vaccination (*46*), LION/repRNA-CoV2S induced ratios of IgG2a:IgG1 greater than 1 (Fig. 3C, D) in all age groups of BALB/c mice, indicating a Th1-biased immune response. Given that severe, life-threatening COVID-19 appears to be more common among elderly individuals, irrespective of type of T helper cell response, and that severe SARS is associated with skewing toward Th2 antibody profiles with an inadequate Th1 response (*16*, *17*, *43*), the ability of LION/repRNA-CoV2S to induce strong Th1-biased responses in 8- and 2-month old mice, even in the Th2-biased BALB/c strain, is potentially a promising finding.

### Safety and immunogenicity of LION/repRNA-CoV2S in pigtail macaques

**Fig. 4:**
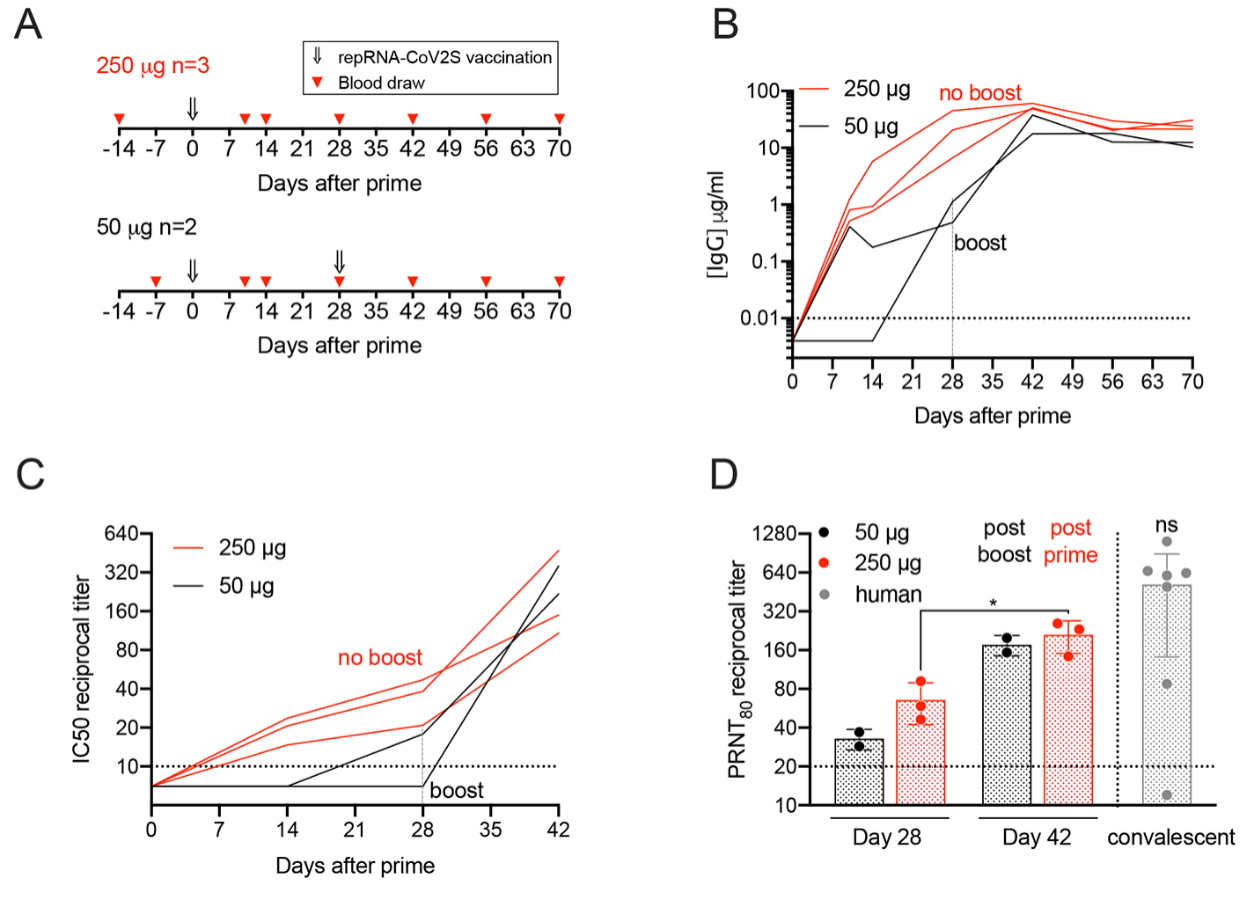
LION/repRNA-CoV2S induces neutralizing antibody responses in pigtailed macaques. (**A**) Pigtail macaques were vaccinated with 250 μg (n=3) or with 50 μg (n=2) LION/repRNA-CoV2S via the intramuscular route and serum was collected on days 10, 14, 28, 42, 56, and 70. The 50 μg dose group received a boost vaccination on day 28 and blood was collected 14, 28, and 42 days later. (**B**) Using pre-immunization blood draws to establish a baseline, serum anti-S IgG enzyme linked immunosorbent assays (ELISAs) were performed on the post-immunization serum samples. (**C**) Pseudovirus (SARS-CoV-2 Wuhan-Hu-1 pseudotype) neutralization assays were performed on serum samples collected on days 14, 28, and 42 to determine mean 50% inhibitory concentrations (IC50) of each sample. Additionally, (**D**) 80% plaque-reduction neutralizing antibody titers (PRNT_80_) against the SARS-CoV2/WA/2020 isolate were measured at days 28 and 42 alongside 7 human convalescent serum samples collected from confirmed COVID-19 patients (see Table S1). The experiment was performed once. Each line in **B** and **C** represents each individual animal. Data in **D** are reported as individual values as well as mean ± s.d. *p<0.05 as determined by students *t* test comparing 250 μg dose groups at days 14 and 28. There was no significant difference (ns) between mean PRNT_80_ titers in all 5 animals at day 42 and titers in sera from 7 convalescent humans, as measured by Mann-Whitney U test.

Having achieved robust immunogenicity with LION/repRNA-CoV2S in mice, we then immunized pigtail macaques (*Macaca nemestrina*) to determine if the vaccine was capable of inducing strong immune responses in a nonhuman primate model that more closely resembles humans in the immune response to vaccination. Three macaques received a prime-only LION/repRNA-CoV2S intramuscular 250 μg dose, administered in 5 injection sites, at week 0 and two macaques received a 50 μg prime dose at week 0 and a boost at week 4, administered into a single intramuscular injection site (Fig. 4A). Blood was collected 10, 14, 28, 42, 56, and 70 days post vaccination to monitor vaccine safety and immunogenicity. The animals were observed daily by veterinary staff for appetite, urine/fecal output and quality, attitude/activity, and no abnormalities were observed. Additionally, the vaccine injection sites were monitored daily for evidence of skin erythema, swelling, discharge, rash, and ulceration for 14 days post vaccination, and no adverse reactions were observed in any of the animals at the injection sites. Furthermore, there were no abnormalities in weight or temperature in the animals (Fig. S2A-B), and serum chemistries revealed no abnormal findings, except for mild azotemia (mildly elevated blood urea nitrogen and creatinine) in 1 animal at 14 days post vaccination (Fig. S2C). Complete blood counts for all 5 macaques were unremarkable (Fig. S2D).

ELISA analyses (Fig. S3) of sera collected 10, 14, 28, 42, 56, and 70 days after prime immunization showed that all three macaques immunized with the prime-only 250 μg dose seroconverted as early as day 10, with anti-S IgG concentrations continuing to increase in these 3 animals to 48, 51, and 61 μg/ml by day 42, then appearing to plateau through at least day 70 post vaccination (Fig. 4B). Both macaques receiving 50 μg repRNA-CoV2S seroconverted after a single dose but developed lower antibody responses with anti-S IgG concentrations of 1 and 0.5 μg/ml by day 28, compared to 7, 20, and 45 μg/ml in the 250 μg dose group at this same time point (Fig. 4B). However, 14 days after a booster immunization, the 50 μg dose group developed similar concentrations of anti-S IgG (18 and 37 μg/ml) as the 250 μg dose prime-only group at this time point (48, 51, and 61 μg/ml) (Fig. 4A). Additionally, sera from the three macaques immunized with the prime-only 250 μg dose neutralized pseudovirus (SARS-CoV-2 Wuhan-Hu-1 pseudotype) transduction of cells in vitro with reciprocal IC50 titers of 1:38, 1:20 and 1:47 by day 28, with IC50 titers increasing to 1:472, 1:108, and 1:149 by day 42; the 50 μg dose group achieved similar robust IC50 titers only after the booster immunization, reaching pseudovirus IC50 titers of 1:218 and 1:358 by day 42 (Fig. 4C and Fig. S4). Sera collected 28- and 42-days post vaccination were further analyzed for neutralization of wild type SARS-CoV-2/WA/2020 by the 80% plaque reduction neutralization test (PRNT_80_). These data were compared to neutralizing titers in sera from convalescent humans collected 15-64 days following natural infection with SARS-CoV-2 (Fig. S4 and Table S1). A prime-only immunization with 50 and 250 μg of LION/repRNA-CoV2S induced mean PRNT_80_ titers by day 28 postvaccination of 1:32 and 1:66, respectively (Fig. 4D). By day 42, mean PRNT_80_ titers significantly increased to 1:176 after a booster immunization in the 50 μg dose group and to 1:211 in the prime-only 250 μg dose group, (Fig. 4D, p=0.012 for both doses, and Fig. S4). Importantly, all 5 macaques developed PRNT_80_ titers within the same range as titers measured in the seven convalescent humans (<1:20 to 1:1280, collected 15 to 64 days post onset of symptoms) and there was no significant difference in mean neutralizing titers between all 5 vaccinated macaques (1:197) and convalescent humans (1:518) (P=0.27, Fig. 4D, Fig. S4, and Table S1). However, larger group sizes will be needed to confirm this finding.

**Fig. 5:**
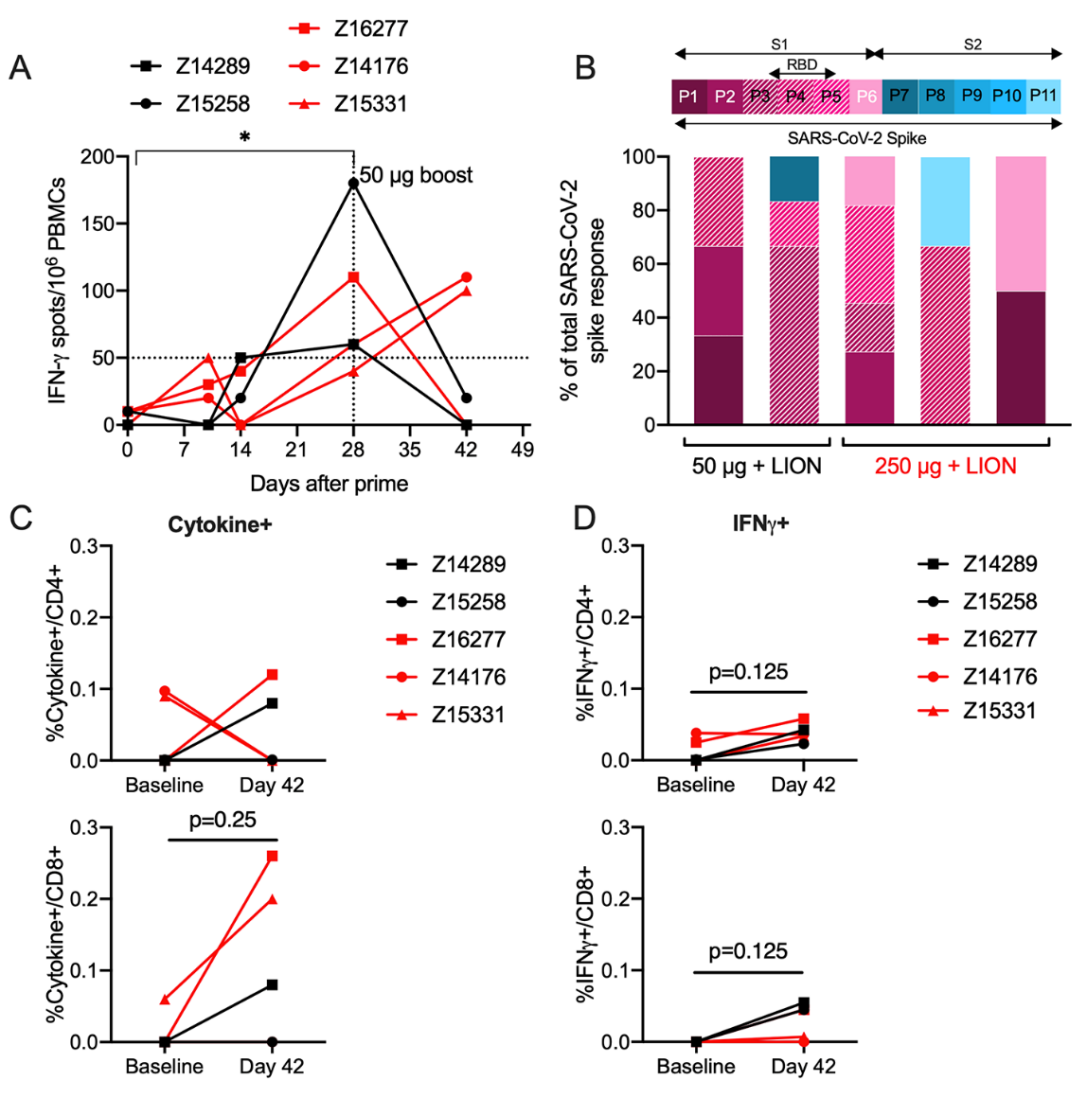
LION/repRNA-CoV2S induces T-cell responses in pigtail macaques. Pigtail macaques were vaccinated with 250 μg (n=3) or with 50 μg (n=2) LION/repRNA-CoV2S via the intramuscular route. PBMCs were isolated from blood at baseline and on days 10, 14, 28, and 42 after prime immunization for T-cell analysis. Shown are (**A**) magnitude and (**B**) breadth of IFN-γ responses measured in PBMCs by ELISpot assay following 24 hour stimulation with 11 peptide pools encompassing the spike (S) protein including the full-length S protein, and S1, S2 and RBD domains. (**B**) Data are presented as percent of total full-length S protein response. (**C, D**). The frequency of S-specific CD4^+^ or CD8^+^ T cells producing any cytokine (IFNγ, IL-2, IL-17A, TNFα, and/or MIP-1β, Granzyme B/CD107a) or IFN-γ alone was determined using cryopreserved PBMCs stimulated overnight with S protein peptides. Shown are the frequencies of S-specific CD4^+^ or CD8^+^ T cells after subtraction of background (DMSO vehicle). Data are from a single experiment. In **A, C, D**, each symbol/line is an individual animal. Data in **B** are representative of each individual animal. **(A)** Friedman test with multiple comparisons, *p<0.05; (**C, D)** Wilcoxon matched-pairs signed rank test; p-values are shown.

ELISpot analyses of peripheral blood mononuclear cells (PMBCs) collected 10, 14, 28 and 42 days post immunization, showed that all 5 macaques developed T cell responses to SARS-CoV-2 S protein by day 28 (Fig. 5A). Peak responses varied between days 28-42 and were independent of the vaccine dose. Similar to findings in our vaccinated mice, the T cell response was directed mostly toward the S1 and RBD regions of SARS-CoV-2 S protein (Fig. 5B). Further evaluation of the T cell response by intracellular cytokine staining revealed modest increases in cytokine-producing CD8^+^ T cells (Fig. 5C and Fig. S5). Frequencies of IFN-γ producing CD4^+^ or CD8^+^ T cells showed an increasing trend in some animals by 42 days post vaccination, although changes were not significant (Fig. 5C). Finally, modest increased frequencies of cytokine or IFN-γ producing S-specific effector memory CD8^+^ T cells were observed by 42 days post vaccination (Fig. 5C,D and Fig. S6).

## DISCUSSION

RepRNA vaccines against a variety of infectious diseases and cancers have been shown to be safe and potent in clinical trials (*47*–*50*). The cell-free and potentially highly scalable manufacturing process for repRNA vaccines when used with effective synthetic formulations, such as LION, present further benefits over mRNA vaccines. The two-vial approach with one vial containing the LION formulation and the other vial containing the repRNA vaccine provides a big manufacturing and distribution advantage over formulations that require complex processes to encapsulate the RNA into lipid nanoparticles within a single vial. Instead, the repRNA vaccine and LION formulation can be scaled-up and stockpiled separately and then combined on site prior to use. Here, we show that the LION/repRNA-CoV2S vaccine induced robust neutralization of SARS-CoV-2 in vaccinated mice and pigtail macaques, with a mean PRNT_80_ titer in all 5 macaques of 1:197. Recently, serum neutralizing titers, measured as the IC50 titer that neutralized SARS-CoV-2 by 50% tissue culture infectious dose (TCID_50_), were reported in rhesus macaques that were either re-infected (*51*) or challenged after vaccination with an inactivated SARS-CoV-2 vaccine (*52*). In the former report, IC50 titers as low as 1:8 were associated with protection from re-infection while in the latter, IC50 titers as low as 1:50 were associated with reduced viral load and protection from lung pathology. In another study, serum neutralizing titers, measured by 50% reduction in luminescence output from a recombinant SARS-CoV-2 virus expressing luciferase, were reported in rhesus macaques that were immunized with a variety of DNA vaccine candidates (*53*) and titers between 1:60 and 1:200 correlated with complete protection from disease. These data suggest that a 250 μg prime-only dose or a 50 μg prime/boost immunization with the LION/repRNA-CoV2S vaccine induced concentrations of neutralizing antibodies that would be sufficient to protect nonhuman primates from infection and disease. Studies are in progress to develop a pigtail macaque SARS-CoV-2 infection model so that this question can be addressed.

Interestingly, the response kinetics in pigtail macaques showed a prolonged increase in serum antibody titers through day 42 and plateauing through at least day 70 post-vaccination. This is most likely due to the sustained antigen expression mediated by repRNA, which may result in plasma cell activation akin to what happens following traditional vaccine booster immunizations. This potential mechanism is currently being investigated. However, because the 250 μg dose was administered concurrently to five injection sites in the pigtail macaques, it cannot be ruled out that immunogenicity and safety measures would be different to those following administration of this dose via a single bolus injection. Studies are ongoing to address this question.

Additionally, we demonstrated that LION/repRNA-CoV2S induces S-specific T cell responses in mice and macaques. Given the relatively recent emergence of SARS-CoV-2, we can only speculate based on limited knowledge from previous and recent reports of coronavirus infection as to how T cell responses may contribute to protection from infection and disease. Following natural infection of humans with the related SARS-CoV, neutralizing antibody and memory B cell responses in some individuals are reported to be short lived (~ 3 years), whereas memory T cells persist for at least 6 years (*54*–*56*), suggesting a potential role for T cells in long-term responses especially in those who lack robust memory B cell responses. Additionally, anti-S T-cell responses to the related SARS- and MERS-CoVs contribute toward viral clearance in normal as well as aged mice infected with SARS- or MERS-CoV, respectively (*43*–*45*). A recent study indicates SARS-CoV-2-specific T cell responses may be associated with better recovery from COVID-19 (*57*). Our findings demonstrate that LION/repRNA-CoV2S elicited modest but detectable T cell responses in macaques (Fig. 5), an outcome that is consistent with previous studies of SARS-CoV-2 S-targeted DNA and live viral vector vaccines tested in nonhuman primates (*53*, *58*). Additionally, the ability of our repRNA vaccine to induce anti-SARS-CoV-2 S-specific memory T cell responses in macaques (Fig. S6) has implications for contributions to long-term protection and recovery from SARS-CoV-2 infection, however, testing of later time points is needed to determine the longevity of this response.

Together, our results demonstrate potential for LION/repRNA-CoV2S, which will enter clinical development under the name HDT-301, to induce rapid immune protection from SARS-CoV-2 infection. A scalable and widely-distributed vaccine capable of inducing robust immunity in both young and aged populations against SARS-CoV-2 infection in a single shot would provide immediate and effective containment of the pandemic. Although our results demonstrate the potential for a prime-only LION/repRNA-CoV2S vaccine (administered over 5 injection sites), only through clinical evaluation of HDT-301 will we know if a single or a prime-boost regimen is required to induce protective immunity in the most optimal and cost-effective way. It is possible that certain demographics, such as the elderly, may require either two doses or a single but higher dose. Critically, the vaccine induced Th1-biased antibody and T cell responses in both young and aged mice as well as pigtail macaques, an attribute that may be important for preventing vaccine-induced immune enhancement of disease. However, these conclusions are drawn in the absence of challenge data and from a limited number of animals and at time points after vaccination where observed responses, especially T-cell responses, exhibited great variability. Additional larger studies will be needed to evaluate safety, kinetics and durability of immune responses, and protection from disease including the absence of immune-enhancement of disease. Together, our results support further development of LION/repRNA-CoV2S as a vaccine candidate for protection against COVID-19.

## MATERIALS AND METHODS

### Study design

The primary objective of this study was to determine and characterize the immunogenicity and safety of a LION/repRNA-CoV2S vaccine in mice and pigtail macaques. Mouse group sizes were based on power analyses using data from previous experiments using a similar repRNA platform and mice were randomly distributed between groups. Group sizes in the macaque study were limited by availability of animals. Five pigtail macaques were stratified based on age and weight. No blinding was used throughout all studies. Endpoints were selected prior to the start of each study and were selected based on the primary objective of characterizing the safety and immune responses to vaccination with a LION/repRNA-CoV2S vaccine. Replication of experiments and the number of biological and technical replicates varied between experiments as described in the figure legends.

### SARS-CoV-2 repRNA vaccine production and qualification

Codon optimized gene sequences for SARS-CoV-2 full S corresponding to positions 21,536 to 25,384 in SARS-CoV-2 isolate Wuhan-Hu-1 (GenBank: MN908947.3) fused to a c-terminal v5 epitope tag was synthesized as double stranded DNA fragments (IDT) and cloned into a plasmid vector encoding the 5′ and 3′ untranslated regions as well as the nonstructural open reading frame of Venezuelan equine encephalitis virus, strain TC-83, between PflFI and SacII sites by Gibson assembly (SGI-DNA). The complete antigen sequence used in repRNA-CoV2S is included in data file S1. Clones were then sanger sequenced and prepped for RNA production as follows. Template DNA was linearized by enzymatic digestion with NotI followed by phenol chloroform treatment and ethanol precipitation. Linearized template was transcribed using MEGAscript® T7 Transcription Kit (Invitrogen) followed by capping with NEB Vaccinia Capping System as previously described (*59*). To qualify the vaccine candidate in vitro, Baby Hamster Kidney (BHK) cells (ATCC) were transfected with repRNA or mock transfected using *TransIT-mRNA* transfection kit (Mirus Bio) and cells analyzed 24 hours later by immunofluorescence using a mouse anti-v5 AF488 secondary antibody (Invitrogen). Additionally, BHK cells were transfected with repRNA-CoV2S and repRNA-GFP and cell lysates were collected 24 hours later for analysis by SDS-PAGE and by Western blot using recombinant SARS-CoV-2 S protein as a positive control. To detect repRNA-mediated protein expression following transfer to nitrocellulose membrane, anti-v5-HRP or convalescent human serum collected 29 days after onset of PCR-confirmed COVID-19 followed by anti-human Ig-HRP secondary antibody (Southern Biotech) was used.

### LION formulation of the repRNA vaccine

To protect the RNA replicons from degradation, we partnered them with a Lipid InOrganic Nanoparticle (LION) formulation that consists of inorganic superparamagnetic iron oxide (SPIO) nanoparticles within a hydrophobic squalene core to enhance formulation stability. LIONs comprise 37.5 mg/ml squalene (Millipore Sigma), 37 mg/ml Span® 60 (Millipore Sigma), 37 mg/ml Tween® 80 (Fisher Chemical), 30 mg/ml DOTAP chloride (Corden Pharma), 0.2 mg/ml 15 nm oleic acid-coated iron oxide nanoparticles (Ocean Nanotech) and 10 mM sodium citrate dihydrate (Fisher Chemical). LION particles were manufactured by combining the iron oxide nanoparticles with the oil phase (Squalene, Span 60, and DOTAP) and sonicating for 30 min in a 65°C water bath. Separately, the aqueous phase, containing Tween 80 and sodium citrate dihydrate solution in water, was prepared with continuous stirring until all components were dissolved. The oil and aqueous phases were then mixed and emulsified using a VWR 200 homogenizer (VWR International) and the crude colloid was subsequently processed by passaging through a microfluidizer at 20,000 psi with a LM10 microfluidizer equipped with a H10Z-100 μm ceramic interaction chamber (Microfluidics) until the z-average hydrodynamic diameter – measured by dynamic light scattering (Malvern Zetasizer Nano S) – reached 50 ±5 nm with a 0.2 polydispersity index. The microfluidized LION was terminally filtered with a 200 nm pore-size polyethersulfone (PES) filter and stored at 2-8°C.

### RNase protection assays

Replicon RNA was complexed with LION formulations and placed on ice for 30 min. After diluting the complex using nuclease-free water, complexes containing 1 μg of repRNA at 20 μg/mL were treated with 50 ng of RNase A (Thermo Scientific) for 30 min at room temperature, followed by an incubation with 5 μg of recombinant Proteinase K (Thermo Scientific) for 10 min at 55°C. RNA was then extracted using an equal volume of 25:24:1 phenol:chloroform:isoamyl alcohol (Invitrogen). After vortexing, samples were centrifuged at 17,000 × *g* for 15 min. The supernatant was collected and mixed 1:1 with Glyoxal load dye (Invitrogen) and heated at 50°C for 15 min. The equivalent of 200 ng of RNA was loaded and run on a denatured 150 mL 1% agarose gel in Northern Max Gly running buffer (Invitrogen) at 120 V for 45 min. Gels were imaged using a ChemiDoc MP imaging system (BioRad). The intensity of the intact RNA band was compared to phenol:chloroform:isoamyl extracted RNA from complexes that were not subjected to RNase and Proteinase K treatment.

### Mouse immunizations

All mouse experiments were conducted in accordance with procedures approved by the institutional animal care and use committee. Female C57BL/6 or BALB/C mice (Charles River) were maintained in specific pathogen-free conditions and entered experiments at 6-12 weeks of age unless otherwise indicated. LION and repRNA-CoV2S were complexed at a nitrogen-to-phosphate molar ratio of 15 in 10mM sodium citrate and 20% sucrose buffer and incubated on ice. Animals were dosed within 30 min after complexing LION and repRNA-CoV2S. All described doses indicate the total quantity of RNA that is formulated and administered to the animals. Mice were immunized by intramuscular injection of vaccine delivered in a total volume of 50 μl in the thigh.

### Pigtail macaque immunizations

Five adult male pigtail macaques were used in these studies (aged 3-6 years, weight 5-13 kg) due to immediate availability of this species of macaque and prior to availability of rhesus macaque SARS-CoV-2 data. All animals received a previous Hepatitis B virus (HBV) DNA and protein vaccine regimen, comprised of HBV core and surface antigens and anti-CD180 (*60*), and were re-enrolled in this study in response to the SARS-CoV-2 pandemic. All animals were housed at the Washington National Primate Research Center (WaNPRC), accredited by the American Association for the Accreditation of Laboratory Animal Care International (AAALAC), and as previously described (*61*). All procedures performed on the animals were with the approval of the University of Washington's Institutional Animal Care and Use Committee (IACUC).

Blood was collected at baseline (week -2 or -1), and at days 10, 14, 28, and 42 post-prime vaccination (Fig. 5A). Blood was also collected 10 days post-boost (38 days post-prime) in the 50μg vaccinated animals. Serum and plasma were collected and PBMCs were isolated from whole blood as previously described (*62*). Animals were sedated with an intramuscular injection (10 mg/kg) of ketamine (Ketaset®; Henry Schein) prior to blood collection or vaccination. Animals were observed daily for general health (activity, appetite) and for evidence of reactogenicity at the vaccine inoculation site (swelling, redness). They also received full physical exams including temperature and weights measurements at each study timepoint. None of the animals became severely ill during the course of the study and none required euthanasia.

Vaccine was prepared as described above. The 50 μg vaccine was delivered intramuscularly into the quadriceps muscle with one 250 μl injection on weeks 0 and 4. To maintain consistency in the vaccine formulation and concentration, the 250μg vaccine was delivered intramuscularly by inoculating 250 μl injections into 5 intramuscular injections sites, 2 in the right quadriceps, 1 in the left quadricep, and 1 each in the left and right deltoids on week 0. All injection sites were shaved prior to injection and monitored post-injection for any signs of local reactogenicity.

### Serum chemistries and complete blood counts

Serum chemistries were run on a Beckman Coulter AU 680/5812 system and complete blood counts were determined on a Sysmex XN9000 analyzer by the University of Washington Department of Laboratory Medicine.

### Antigen-specific antibody responses

Blood was collected from the retro-orbital sinus of immunized mice, or venipuncture of anesthetized macaques, and serum prepared. Antigen-specific IgG, IgG1, IgG2a, and IgG2c responses were detected by enzyme linked immunosorbent assay (ELISA) using a previously described recombinant SARS-CoV-2 S as the capture antigen (*63*). ELISA plates (Nunc) were coated with 1 μg/ml antigen or with serial dilutions of purified polyclonal IgG from mouse or monkeys to generate a standard curve in 0.1 M PBS buffer and blocked with 0.2% BSA-PBS. Then, in consecutive order, washes in PBS/Tween, serially diluted serum samples, anti-mouse or-monkey IgG, IgG1, IgG2a, or IgG2c-HRP (Southern Biotech) and TMB then HCL were added to the plates. Plates were analyzed at 405nm (EL_X_808, Bio-Tek Instruments Inc). Absorbance values from the linear segment of each serum dilution curve was used to interpolate the standard curve and calculate the IgG concentration present in each sample. For IgG isotype-specific ELISAs, endpoint titers were determined using 3 times the standard deviation above the mean of triplicate negative-control sera as the cut-off absorbance value. IgG2c isotype was selected for C57BL/6 sera due to their lack of IgG2a isotype (*64*), while IgG2a is utilized for BALB/C sera (*65*).

### SARS-CoV-2 pseudovirus neutralization assay

Murine leukemia virus (MLV)-based SARS-CoV-2 S-pseudotyped viruses were prepared as previously described (*63*, *66*). In brief, HEK293T cells were co-transfected with a SARS-CoV-2 (based on Wuhan-Hu-1 isolate) S-encoding plasmid, an MLV Gag-Pol packaging construct, and the MLV transfer vector encoding a luciferase reporter using the Lipofectamine 2000 transfection reagent (Life Technologies) according to the manufacturer’s instructions. Cells were incubated for 5 hours at 37°C with 8% CO2 with DNA, lipofectamine, and OPTIMEM transfection medium. Following incubation DMEM containing 10% FBS was added for 72 hours. Pseudovirus was then concentrated using a 30kDa Amicon concentrator for 10 min at 3,000 × g and frozen at -80C.

BHK cells were plated in 96 well plates for 16-24 hours prior to being transfected with human ACE2 using standard lipofectamine 2000 protocol and incubated for 5 hours at 37°C with 8% CO2 with DNA, lipofectamine, and OPTIMEM transfection medium. Following incubation, DMEM containing 20% FBS was added in equal volume to the OPTIMEM transfection media for 16-24 hours. Concentrated pseudovirus with or without serial dilution of antibodies as incubated for 1 hour at room temperature and then added to the wells after washing 3X with DMEM and removing all media. After 2-3 hours, equal volumes of DMEM containing 20% FBS and 2% PenStrep was added to the cells for 48 hours. Following 48 hours of infection, equal volume of One-Glo-EX (Promega) was added to the cells and incubated in the dark for 5-10 min prior to reading on a Varioskan LUX plate reader (ThermoFisher). Measurements were done in duplicate and relative luciferase units (RLU) were recorded.

### Human convalescent sera

De-identified remnant diagnostic samples from individuals with COVID-19 in Washington state were obtained via Northwest Biospecimens (Seattle, WA) following registration for exemption from IRB approval with the University of Washington Human Subjects Division.

### SARS-CoV-2 neutralization assay

Three-fold (pigtail macaque) or four-fold (human) serial dilutions of heat inactivated serum and 600 plaque-forming units (PFU)/ml solution of SARS-CoV-2/WA/20 (BEI resources) were mixed 1:1 in DPBS (Fisher Scientific) + 0.3% gelatin (Sigma G7041) and incubated for 30 min at 37°C. Serum/virus mixtures were added in duplicate, along with virus only and mock controls, to Vero E6 cells (ATCC) in a 12-well plate and incubated for 1hr at 37°C. Following adsorption, plates were washed once with DPBS and overlayed with a 1:1 mixture of Avicel RC-591 (FMC) + 2 x MEM (ThermoFisher) supplemented with 4% heat-inactivated FBS and Penicillin/Streptomycin (Fisher Scientific). Plates were then incubated for 2 days at 37°C. Following incubation, overlay was removed and plates were washed once with DPBS and then 10% formaldehyde (Sigma-Aldrich) in DPBS was added to cells and incubated for 30 min at room temp. Plates were washed again with DPBS and stained with 1% crystal violet (Sigma-Aldrich) in 20% EtOH (Fisher Scientific). Plaques were enumerated and percent neutralization was calculated relative to the virus-only control.

### Mouse IFN-γ ELISpot assay

Spleen and lung lymphocytes were isolated from mice 12 days after the second vaccination. MIAPS4510-Multiscreen plates (Millipore) were coated with rat anti mouse IFN-gamma capture antibody (BD) in PBS and incubated overnight at 4°C. The plates were washed in PBS and then blocked (2h, RT) with RPMI medium (Invitrogen) containing 10% heat inactivated fetal calf serum (Gibco). Lung and spleen cells were plated at 5x10^5^ and 2.5x10^5^ cells/well and stimulated with the SARS-Cov2 S peptide pools (11aa overlapping 15 mer peptides from Genscript) at 1.5ug/ml/peptide and cultured for 20 hours (37°C, 5% CO_2_). Biotinylated anti-mouse IFN-gamma antibody (BD) and streptavidin-Alkaline Phosphotase-substrate (Biolegend) were used to detect IFN-gamma secreting cells. Spot forming cells were enumerated using an Immunospot Analyzer from CTL Immunospot profession software (Cellular Technology Ltd).

### Macaque IFN-γ ELISpot assay

PBMCs were stimulated for 24 hours with SAR-CoV-2 Spike (Genscript) as described above including 17- or 18-mers with 11 amino acid overlap, peptide pools at a final concentration of 1μg/mL. Concanavalin A (Thermofisher) 2.5 μg/mL was used as a positive control, and DMSO at a concentration equal to peptide stimulations was used as a negative control. Antigen-specific cells secreting IFN-γ were detected using an Immunospot Human IFN-γ Single-Color Enzymatic ELISPOT Assay, per manufacturer’s protocol. Spot forming cells (SFC) were enumerated as described above and results were considered positive if the number of SFC was greater than that of the negative control and for 1x10^5^ cells was ≥ 1 per well.

### Macaque cell intracellular cytokine staining

Multiparameter flow cytometry was used to determine T-cell immune responses using peptide stimulated PBMC as previously described (61). Cells were stained with the following antibodies: Live/Dead Aqua (Invitrogen®, Catalog #:L34957), CD4 BV605 (BioLegend, clone OKT4), CD3 BV650 (BD Biosciences, clone Sp34-2), CD8 BV786 (BioLegend, clone RPA-T8), CD45 PECF594 (BD Biosciences, clone D058-1283), CD95 APC-Cy7 (BioLegend, clone DX2), CD28 BUV395 (BD Biosciences, clone CD28.2), CCR7 BUV295 (BD Biosciences, clone CD28.2), (CD107a PeCy5 (eBioscience, clone 3D12), IL-2 AF700 (BioLegend, clone MQ1-17H12), IFNg FitC (BD Biosciences, clone B27), MIP-1β PerCp-Cy5.5 (BD Biosciences, clone D21-01351), Granzyme B BV421 BD Biosciences, clone GB11), TNFα Pe-Cy7 (BD Biosciences, clone MAb11), and IL-17 PE (eBioscience, clone eBio64CAP17). Values of peptide-stimulated cells are after negative control (DMSO) subtraction. All cells were fixed in 1% paraformaldehyde and acquired using an LSR II flow cytometer (BD Biosciences) and analyzed using FlowJo software (Version 10.6.2, Tree Star, Inc.). To quantify the frequency of Cytokine^+^ T cells producing IFNγ, IL-2, IL-17A, TNFα, and/or MIP-1β, Granzyme B/CD107a, an ‘or’ Boolean Gating strategy was utilized in FlowJo. The displayed frequency of Cytokine^+^ T cells after peptide stimulation was determined by subtracting Boolean gated Cytokine^+^ T cell frequency in DMSO negative controls (Supp Fig. 5).

### Statistical analyses

Statistical analyses were conducted in Prism (Graphpad) using one-way analysis of variance and Tukey’s multiple comparison test to compare more than two groups. Student’s *t* test, Mann Whitney U test, or Wilcoxon rank sum test were used to compare two groups. Statistical significance was considered when the *p*-values were < 0.05.

## Supplementary Materials

stm.sciencemag.org/cgi/content/full/scitranslmed.abc9396/DC1 Fig. S1. Breadth of T cell responses in C57BL/6 and BALB/c mice. Fig. S2. Vaccination did not induce adverse reactions in pigtail macaques. Fig. S3. Raw ELISA absorbance values for pigtail macaques. Fig. S4. Neutralization curves for pigtail macaque and human samples against SARS-CoV-2/WA/2020 or pseudotyped virus. Fig. S5. Gating strategies used for evaluation of peptide-specific intracellular cytokine staining. Fig. S6. LION/repRNA-CoV2S induces memory T-cell responses in pigtail macaques. Table S1. Convalescent sera from individuals with COVID-19. Data file S1. Source data for all figures.
